# Actively evolving subglacial conduits and eskers initiate ice shelf channels at an Antarctic grounding line

**DOI:** 10.1038/ncomms15228

**Published:** 2017-05-09

**Authors:** R. Drews, F. Pattyn, I. J. Hewitt, F. S. L. Ng, S. Berger, K. Matsuoka, V. Helm, N. Bergeot, L. Favier, N. Neckel

**Affiliations:** 1 Université libre de Bruxelles, Laboratoire de Glaciologie, Avenue F.D. Roosevelt 50, Brussels 1050, Belgium; 2 Bavarian Academy of Sciences and Humanities, Glaziologie, Alfons-Goppel-Str. 11, Munich 80539, Germany; 3 University of Oxford, Mathematical Institute, Woodstock Road, Oxford OX26GG, UK; 4 The University of Sheffield, Department of Geography, Winter street, Sheffield S102TN, UK; 5 Norwegian Polar Institute, Fram Centre 9296 Tromsø, Norway; 6 Alfred Wegener Institute, Am Alten Hafen 26, Bremerhaven 27568, Germany; 7 Royal Observatory of Belgium, Av. Circulaire 3, Brussels 1180, Belgium

## Abstract

Ice-shelf channels are long curvilinear tracts of thin ice found on Antarctic ice shelves. Many of them originate near the grounding line, but their formation mechanisms remain poorly understood. Here we use ice-penetrating radar data from Roi Baudouin Ice Shelf, East Antarctica, to infer that the morphology of several ice-shelf channels is seeded upstream of the grounding line by large basal obstacles indenting the ice from below. We interpret each obstacle as an esker ridge formed from sediments deposited by subglacial water conduits, and calculate that the eskers' size grows towards the grounding line where deposition rates are maximum. Relict features on the shelf indicate that these linked systems of subglacial conduits and ice-shelf channels have been changing over the past few centuries. Because ice-shelf channels are loci where intense melting occurs to thin an ice shelf, these findings expose a novel link between subglacial drainage, sedimentation and ice-shelf stability.

## Abstract (web summary)

The formation mechanisms of ice-shelf channels remain poorly understood. Here, using ice-penetrating radar data, the authors propose that ice-shelf channel morphology in the Roi Baudouin Ice Shelf, East Antarctica, is seeded by esker ridges, indenting the ice from below.

Water beneath the Antarctic Ice Sheet promotes the formation of ice streams that rapidly slide over wet sediments and a lubricated base. Ice streams discharge the majority of Antarctic ice into floating ice shelves, which surround about 74% of the Antarctic perimeter^1^. Ice shelves occupying embayments buttress the continental mass flux^2^. The buttressing strength depends on the pattern of basal mass balance (i.e., the sum of melting and refreezing), which in turn influences ice-shelf geometry^3^. Measurements show that basal melting is concentrated by ice-shelf channels^4^,^5^,^6^, which are typically a few kilometres wide and extend for up to hundreds of kilometres along the shelf flow. Ice is thinnest along their central axes (sometimes thinner than half of the ice thickness^7^), and basal melt rates are elevated at their onsets near the grounding line^6^. Theory and satellite-based observations suggest that such ‘subglacially sourced' ice-shelf channels^8^ are formed by buoyant melt-water plumes forced by basal melt water exiting from subglacial conduits at the grounding line^9^,^10^. Hitherto, no such conduits have been observed, presumably because they are too small to be detected with ice-penetrating radar^10^.

In this study we use satellite data and ice-penetrating radar to show that ice-shelf channels on the Roi Baudouin Ice Shelf, East Antarctica, are seeded upstream of the grounding line by basal obstacles indenting the ice from below. These obstacles align with predicted hydrological outlets, and thus we interpret them as eskers (sediment ridge composed of gravel and sand) formed by the overlying subglacial water conduits. Our findings confirm a recognized linkage between ice-shelf channel formation and subglacial hydrology^6^,^9^,^10^. However, we show that much of an ice-shelf channel's amplitude can be created upstream of grounding line where the ice overrides an esker. Existing theories of ice-shelf channel development from basal topographical undulations^11^,^12^ have not considered this possibility. Our analysis, therefore, provides a novel link between ice-shelf buttressing and sedimentation, as well as evidence of eskers beneath a contemporary ice sheet.

## Results

### Overview

**Figure 1:**
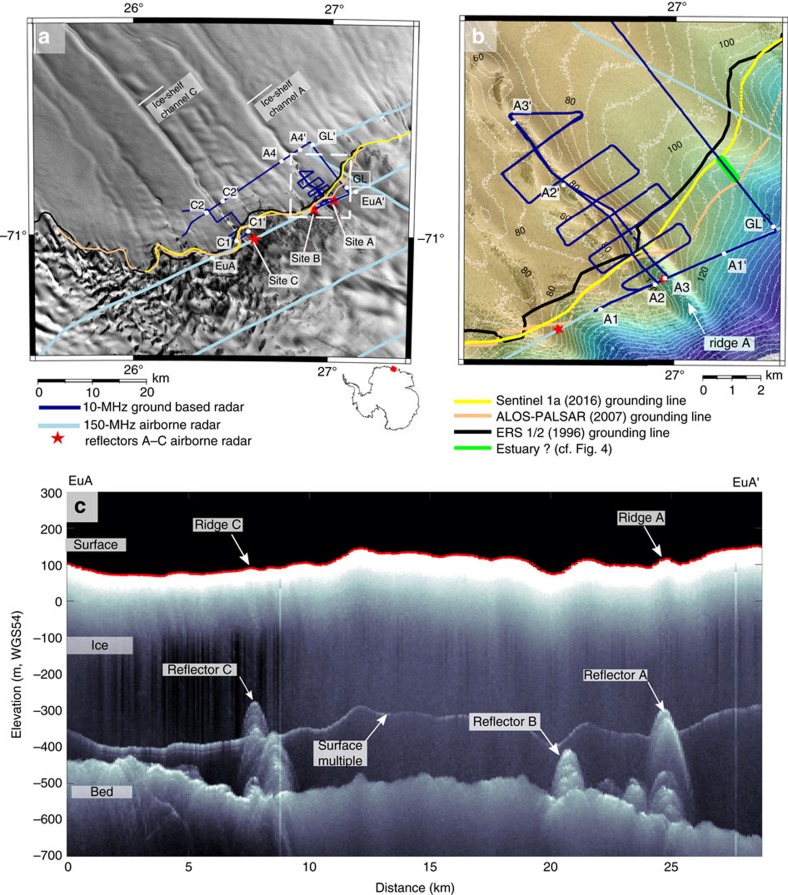
Overview of the study area. (**a**) Location of airborne (2011) and ground-based (2016) radar profiles of the Roi Baudouin Ice Shelf, East Antartica, with Landsat image in the background. Grounding lines are marked for 1996, 2007 and 2016. The dashed white box delineates the area in **b** where radar-profile locations are shown with TanDEM-X surface elevation (5 m contours). (**c**) Airborne radar profile EuA-EuA′ covering the grounded ice sheet. Internal reflection hyperbolas reaching hundreds of metres above the ice-bed interface are evident (reflectors A–C), and are aligned with ice-shelf channels located seawards (into page). Reflectors A and C are beneath surface ridges.

**Figure 2:**
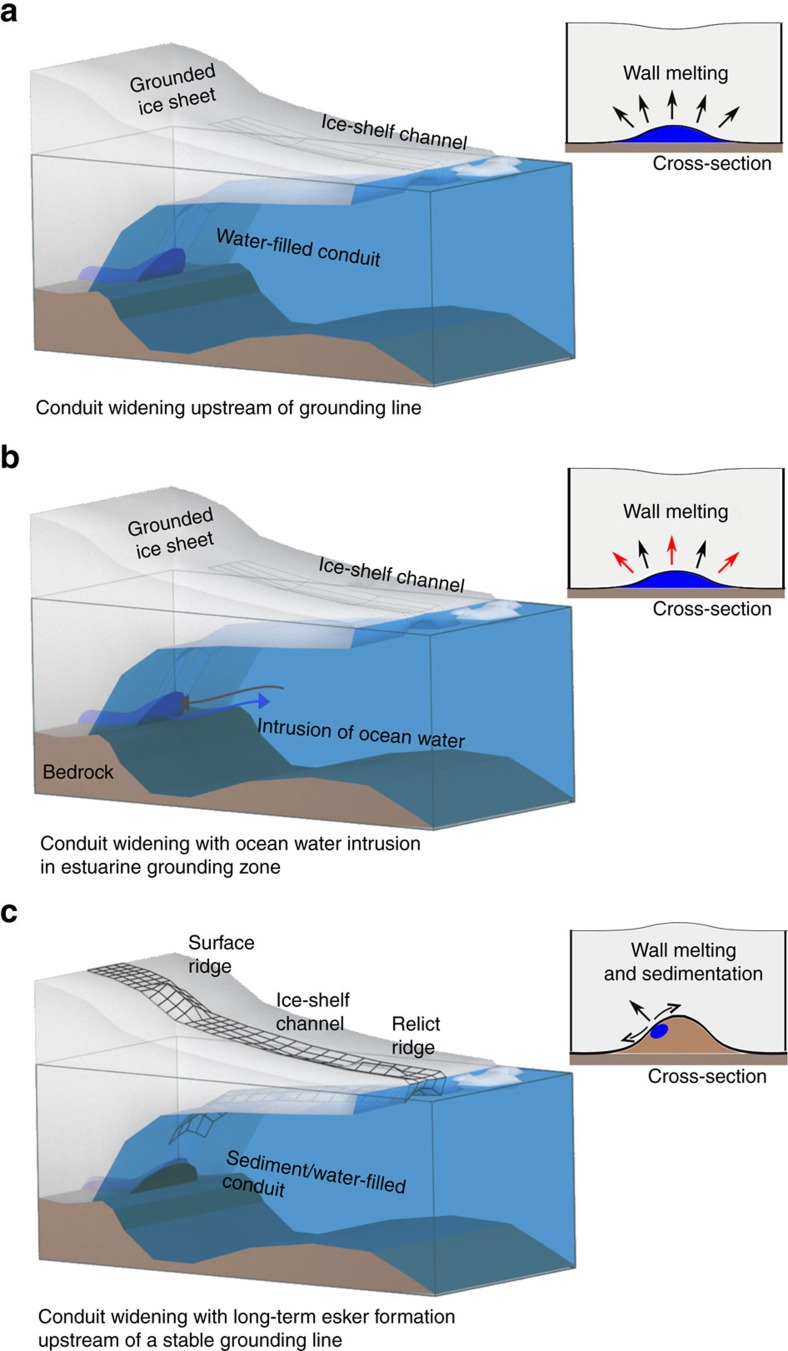
Hypothesis for ice-shelf channel formation by subglacial water conduits. (**a**) Subglacial water conduits widen close to the grounding line due to diminishing closure rate forming an ice-shelf channel seawards. (**b**) Conduit widening is further amplified by intrusion of warmer ocean water, and (**c**) sedimentation forms an esker at the conduit's portal as a consequence of conduit widening and decreasing water outflow speed. Ice flow over the esker causes a ridge to form at the surface, which may be preserved as a relict ridge in the ice shelf. This scenario accords best with our observations.

Here we survey three hydrologically predicted^10^ subglacial water-outlet locations at the Roi Baudouin Ice Shelf in Dronning Maud Land, Antarctica, all with corresponding ice-shelf channels seawards (Sites A–C, Fig. 1a,b). Airborne radar data collected upstream of the satellite-inferred grounding line show distinct radar reflectors situated several hundred metres above the adjacent ice-bed interface (reflectors A–C, Fig. 1c). Using additional ground-based radar data from 2016, we examine the reflectors' geometry in order to deduce their identity and evaluate three different scenarios for ice-shelf channel formation (Fig. 2): (1) the reflectors are the top surfaces of subglacial water conduits (thus, local upwarpings of the ice-bed interface) that widen towards the grounding line, and this basal morphology seeds the ice-shelf channels, (2) same as (1), but conduit widening is further amplified by the intrusion of warmer ocean water and (3) the reflectors are large, ridge-shaped basal obstacles protruding up into the ice flow that generate the initial ice-shelf channel morphology.

In full details below, we argue that scenario 3 accords best with our observations, and we interpret each basal obstacle as an actively evolving ramp-shaped esker whose size increases towards the ocean due to subglacial conduit widening and decreasing water flow speed. Eskers, a glacial landform used in the reconstruction of palaeo ice sheets^13^, are the depositional evidence of former channelized subglacial hydrological systems^14^,^15^. Our inferred eskers are much larger than most eskers of the Wisconsinan glacial record, but, as described later, their shape resembles that of some eskers in deglaciated areas formerly occupied by marine-terminating ice sheets^16^,^17^.

### Location and geometry of reflectors A to C

**Figure 3:**
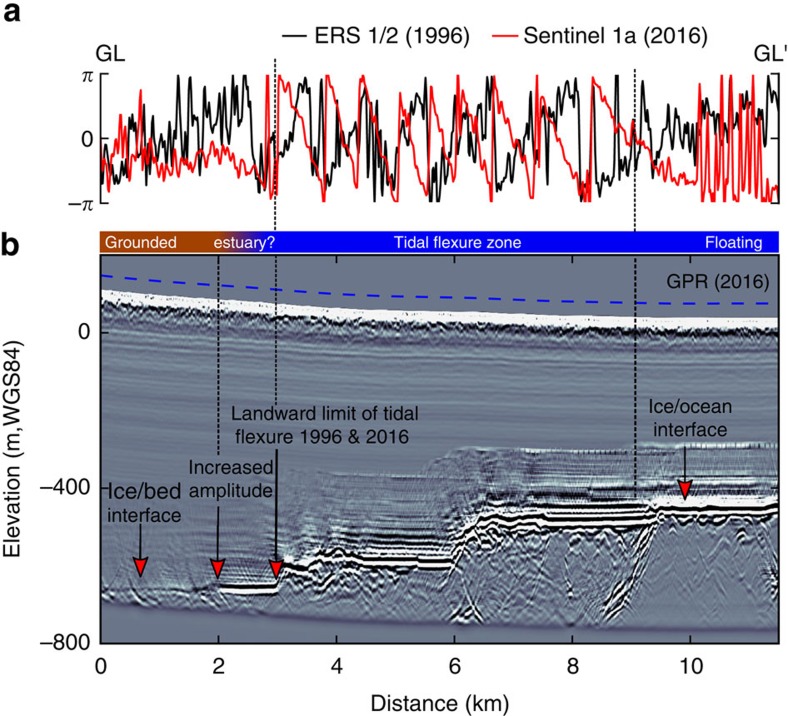
Characterization of the grounding zone. Satellite radar interferometry from 1996 and 2016 (**a**) together with ground-penetrating radar (GL-GL′ located in Fig. 1) (**b**). Radar reflection amplitudes suggest basal water upstream of the tidal flexure limit, potentially indicating an estuary with intrusion of ocean water. The blue dashed curve delineates the surface.

We estimate the grounding-line position using satellite-based interferometric synthetic aperture radar by picking the landward limit of the tidal flexure zone in interferograms from 1996, 2007 and 2016 (Supplementary Note 1 and Supplementary Fig. 1). The limit moved negligibly between these years and suggests that reflectors A–C have been located up to 1.5 km upstream of the grounding line for at least two decades. Stability of the sheet-shelf system on millennial time scales in this area is supported by the long ice-divide residence of an ice rise located in the ice shelf^18^, and by a modelling study showing that the grounding line has a strong topographic control hampering its retreat even in high basal melt scenarios^19^. We therefore rule out the interpretation that reflectors A–C are relict ice-shelf channels formed some time before 2016 when the grounding line had receded, and the grounding line subsequently advanced. This interpretation is also inconsistent with the ice-surface ridges above the reflectors mentioned below, because a depression rather than ridge would form above a large basal channel. Our ground-based radar profiles corroborate the satellite-based grounding-line positions and also indicate basal water upstream of the tidal flexure zone (Fig. 3). This water may be of continental origin or signify an estuarine grounding zone where ocean water penetrates upstream of the tidal flexure zone through tidal pumping^20^,^21^,^22^.

**Figure 4:**
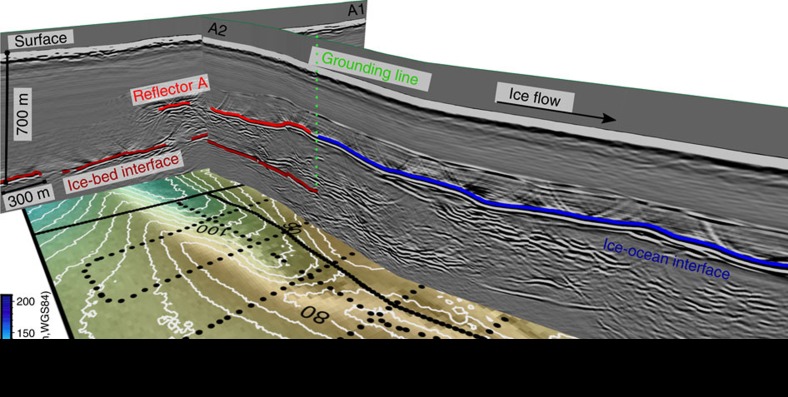
3D view of ice-shelf channel formation. Perspective view of migrated, ground-based radar profiles A1-A1′ and A2-A2′ and interpretations of reflection interfaces (ice-bed, ice-ocean and reflector A). The bottom image delineates additional ground-based radar profiles (black dots) together with TanDEM-X surface elevation.

**Figure 5:**
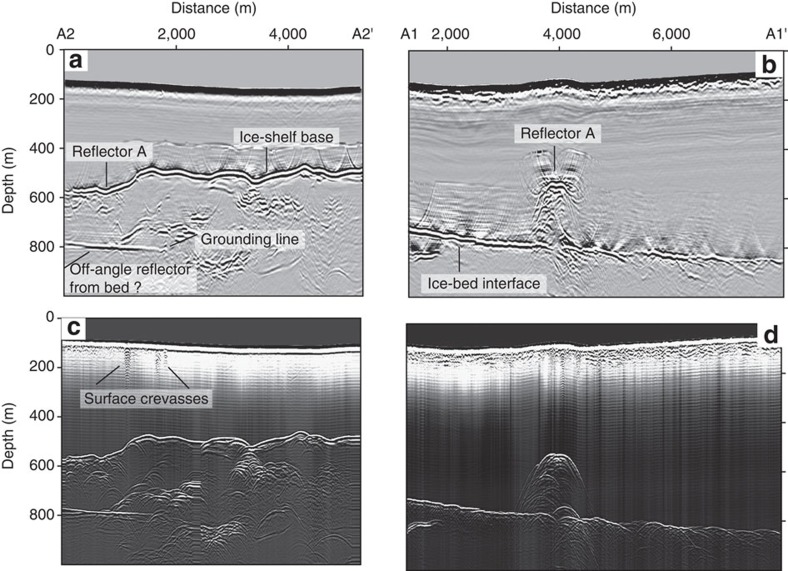
Plain view of ice-shelf channel formation. 2D representation of the radar profiles A1–A1′ and A2–A2′ with (**a**,**b**) and without (**c**,**d**) migration. Depth conversion for (**c**,**d**) uses standard normal-moveout correction^58^. The migrated along-flow section (**a**) shows the location of the grounding line, and the transition from the top of reflector A into the base of the floating ice shelf. Reflections from near-surface crevasses seen in (**c**) are potentially caused by tidal bending. In the across-flow section (**d**), the reflection hyperbolas of reflector A collapse during the migration (**b**) showing that lateral walls are too steep to be imaged by our nadir-looking radar.

**Figure 6:**
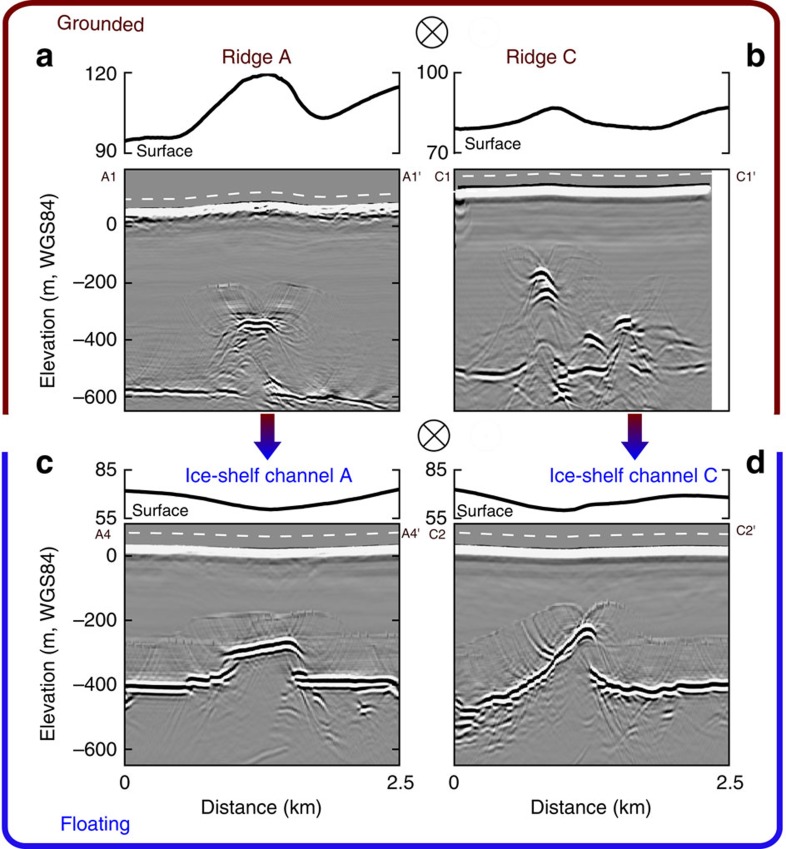
Surface ridges landwards of ice-shelf channels. (**a**,**b**) Migrated radargrams, aligned across-flow, showing the reflectors A and C located on grounded ice. The reflectors turn into ice-shelf channels (**c**,**d**) incising the floating ice shelf. The top panels display the surface topography in which the grounded sections both show prominent surface ridges aligned with the respective reflector locations. In the floating ice-shelf channels, the ridges are mostly absent, and the surfaces are depressed. The white dashed curve in the radargrams marks the surface.

Reflectors A–C may arise from localized inhomogeneities within the ice or indicate upwarpings of the ice base, e.g., they image the roof of volumes of non-ice material above the bed that extend longitudinally. To distinguish between these possibilities, we examine the reflectors' geometry and orientation at site A using ground-based radar profiles that have been migrated. (The unmigrated airborne profile in Fig. 1c is unsuitable for this purpose.) Radar profile A1–A1′ (Figs 4 and 5) shows that reflector A spans 330 m across the ice flow. The cross-section A2–A2′ links reflector A in the along-flow direction for 1.8 km to the grounding line and farther into the ice-shelf (Fig. 4). Our complete set of gridded profiles determines the horizontal dimensions (∼300 × 1,800 m^2^) of reflector A upstream of the grounding line. We see no reflections delineating lateral walls, so either no walls exist or they are too steep to be imaged by our nadir-looking radar. The former interpretation here implies an essentially two-dimensional internal reflector, which is inconsistent with the seaward extension of these features into ice-shelf channels where ice is lacking compared to the neighbouring areas (Fig. 6). We, therefore, conclude that our gridded radar data image the roof of a subglacial disruption of the ice-bed interface which is up to 250 m high, about 300 m wide and at least 1.8 km long. About 15 km farther upstream, no such feature can be seen in the airborne data (Supplementary Fig. 2) so the disruption decays with distance upstream of the grounding line.

The airborne radar, and to a lesser extent the ground-based radar, show additional reflections below reflectors A and C (Figs 1 and 6a,b), which may arise from internal heterogeneities or off-angle reflections from the heavy crevassing in this area^23^. Reflectors A–C have the same phase as the emitted wave, indicating a transition from an upper, optically less dense material to a lower, optically denser material. This excludes an ice–air interface, but does not distinguish whether the lower medium is water or sediment. The co-location of reflectors A–C with water-outlet positions calculated from the hydrostatic potential field of the upstream ice-flow catchment^10^ (Fig. 1c in ref. 10), however, indicates an active role of subglacial hydrology in the origin of the interfacial upwarpings.

**Figure 7:**
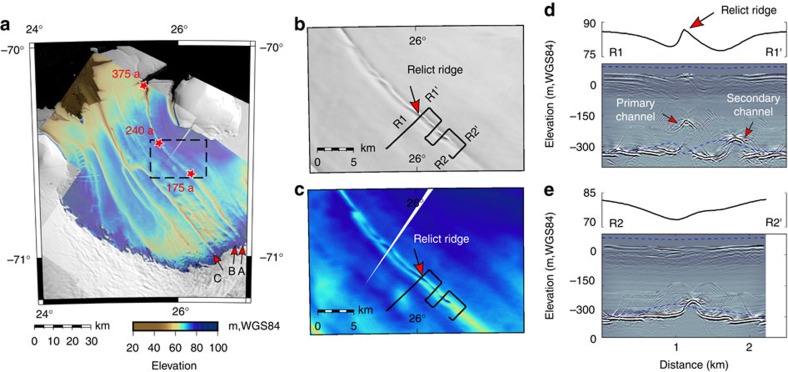
Relict surface ridges within ice-shelf channels. (**a**) TanDEM-X surface elevation illustrating the surface depression corresponding to ice-shelf channels. Advection times of present-day velocities are marked with red stars. The black box is enlarged in (**b**) where the Landsat image shows an ice-shelf channel being subdivided by a surface ridge (**c**). This ridge is incised by a primary channel accompanied by a secondary channel (**d**). Farther upstream (R2–R2′, (**e**), the ice-shelf channel is more typical consisting of one surface depression and one basal incision. The blue lines correspond to the hydrostatic ice thickness and show that the surface ridge and the ice-shelf channel are not in hydrostatic equilibrium.

Our ground-based kinematic GNSS data and the surface elevation model show that surface ridges exist above reflectors A and C. These are ∼200–400 m wide, ∼10–30 m high and up to 3 km long (Figs 1b and 6). A surface ridge is visible also above reflector B, with its crest offset laterally from the reflector. On the shelf surface a few kilometres downstream of the grounding line, some dispersed hills occur within the surface depressions of ice-shelf channels A and C (e.g., site A2′ in Fig. 1b); also, 45 km farther downstream, there is a sinuous, 10 km long surface ridge inside ice-shelf channel A (Fig. 7a,b). We will discuss the causes of these topographic features after addressing scenarios 1–3 in the next section.

### Widening of subglacial conduits near the grounding line

The most striking features of our observations are large upward disruptions of the ice-bed interface upstream of ice-shelf channels. They are orders of magnitudes larger than the cross-sections of typical subglacial conduits, which are expected to be a few metres in diametre^10^. Their diminishing amplitude upstream implies that their formation is due to processes near the grounding line.

**Figure 8:**
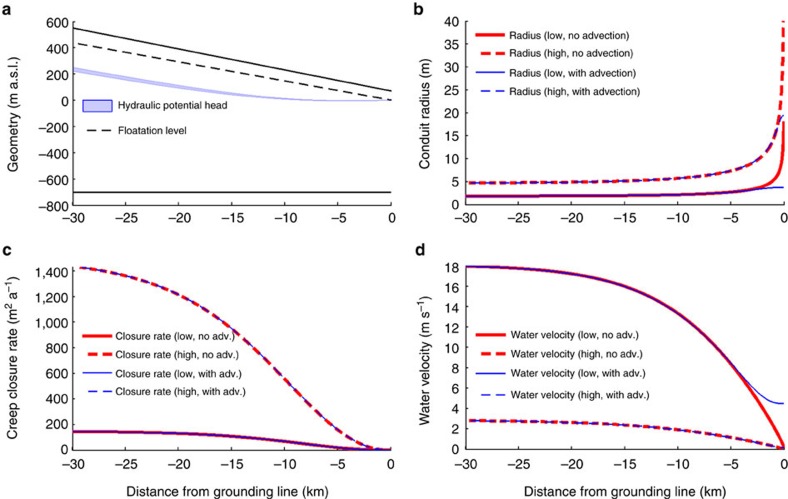
Hydrological model for conduit-widening upstream of the grounding line. Steady-state solution for Röthlisberger channels upstream of the grounding line. We present a lower (low: discharge *Q*=10 m^3^ s^−1^, basal ice velocity *u*_b_=300 ma^−1^) and an upper (high: *Q*=100 m^3^ s^−1^, *u*_b_=1 ma^−1^) scenario for the conduit cross-sections without (red) and with (blue) including ice advection. (**a**) Shows hydraulic potential (independent of ice advection) represented as metres of head in blue. Solid black line represents the ice surface elevation and dashed line represents the flotation level. (**b**) Shows radius of the conduit (without advection the radius tends to infinity at the grounding line). (**c**) Shows creep closure rate which equals the conduit-wall melting rate except close to the grounding line. (**d**) Shows the water velocity in the channel.

We first examine scenario 1 (Fig. 2a) using the concept of Röthlisberger channels, which we modify to incorporate the grounding line and ice advection. Röthlisberger channels are located at the ice-bed interface and incise the ice from below with a roughly semicircular shape^24^,^25^. Their operation can be understood as a competition between wall melting (the heat being provided by turbulent water flow) and creep closure due to overburden ice pressure. At the grounding line, ice is close to hydrostatic equilibrium and water pressure inside the conduit must equal the ocean pressure. Because of this balance, effective pressure and hence creep closure rate are zero. Melting at the channel walls, on the other hand, persists there, even though melt rate decreases as the cross-section widens. Without a closing mechanism, the steady-state conduit cross-section grows infinitely large over time. However, ice flow adds an advective component of thicker ice from upstream which keeps the cross-section finite. We employ a numerical model (Supplementary Note 2) to quantify the conduit widening at the grounding line, and investigate the impact of a range of parameters (i.e., the discharge and basal ice velocity). We use a hybrid ice sheet/ice stream model^26^ to estimate the subglacial meltwater production for the Roi Baudouin catchment area using the most recent bed topography^27^. Most meltwater originates from frictional heat at the ice-bed interface and the integrated flux across the grounding line is ∼60 m^3^ s^−1^. Figure 8a illustrates the simplified ice sheet defining the hydrostatic potential. The corresponding conduit has a constant radius (∼2–5 m) for about 25 km, and then widens (∼2.5–20 m) in a narrow band 2–5 km upstream of the grounding line. Creep closure rate and water velocity also decrease, the former more gradually than the latter (Fig. 8c,d). Although conduits are predicted to widen up to four-fold, they are too small to explain the height of the observed disruptions. Thus scenario 1 cannot fully explain our observations.

### Widening of subglacial conduits and ocean water intrusion

Our radar data (Fig. 3) gives some evidence that ocean water may penetrate upstream of the tidal flexure through tidal pressure variations^20^, which has been observed in some locations of the Whillans Ice Stream grounding zone^21^,^22^. In our case, ocean water may intrude into the conduit causing stratification of the subglacial water on top of the heavier, saline ocean water (Fig. 2b). In this way, additional heat can be entrained from the ocean through a fresh-water plume, resulting in higher melt rates at the conduit walls than would occur through turbulent dissipation alone. Continuous melting is required to maintain the large cross-sections, which will otherwise close through the advection of thicker ice. Based on the local surface velocities of about 300 m a^−1^ (ref. 28), we estimate that melt rates of ∼10 m a^−1^ are required to keep the observed cross-section in steady state (Supplementary Note 2). This is an upper limit, because basal velocities are likely smaller than surface velocities^29^. Such scenario has been described before^9^,^10^, and observations in Greenland testify to the potential for basal melting of fresh-water plumes which cause undercutting of marine-terminating glaciers^30^,^31^. However, conduit widening and intrusion of ocean water alone do not explain the surface ridges that are located above the reflectors A and C (Figs 1c and 6). On the contrary, continuous melting inside conduits will only lower the ice surface. We, therefore, rule out scenarios 1 and 2 as a sole mechanism for ice-shelf channel formation and investigate next scenario 3 (Fig. 2c) that includes a basal obstacle indenting the ice from below.

### Basal channel formation by subglacial conduits and eskers

In scenario 3 (Fig. 2c), large basal obstacles are envisaged to be the cause of both upwarping of the ice base and the surface ridges. The key considerations here are how such bedforms arise, and their relationship with subglacial water conduits.

Subglacial conduits can erode material where the bed is deformable, and incise into the ice where the bed is hard^21^,^32^,^33^,^34^,^35^. Sediment outwash from subglacial conduits has been observed at marine-terminating ice margins in Svalbard^36^ and for an outlet glacier in Greenland^37^. Although it is non-trivial to model the corresponding sediment transport rates in a reliable manner (due to uncertain constraints on till deformation and scarcity of direct subglacial measurements), we can qualitatively consider the effects of conduit widening in such systems. As the conduit widens downstream, subglacial water flow speed decreases (Fig. 8b), reducing its sediment transport capacity. This causes sediment to deposit near the conduit's portal. No rigorous framework exists for predicting the response of a marine-terminating conduit to the resulting accreting bedform, but geomorphologists have suggested that tunnel sedimentation enhances melting at the conduit's roof and that the bedform can cause hydraulic feedback to sustain sediment deposition, thereby furthering its own growth^14^,^33^,^38^. Over time, this process can create a sharp-crested esker, with one or more subglacial conduits (whose size is smaller than the esker) wandering along its upper ice–sediment boundary and continuing to incise upward (ref. 16 Fig. 828 on p. 239 and ref. 39). This scenario matches our observations well because the esker would be ramp-shaped, several kilometres long (deduced from the typical length-scale over which water velocity drops; Fig. 8), and have mechanical contact with the overriding ice flow so that its shape causes a surface topographic ridge to form^40^. The initial bottom topography of an ice-shelf channel is then moulded at the grounding line. Because the esker evolves actively in this coupled system, complete blockage of the conduit by sediments may eventually occur, forcing subglacial water to reroute or flush the sediments in an outburst flood^13^,^41^,^42^. An alternative, but much less convincing, explanation for our observations is that reflections A–C originate from other protruding bedforms such as drumlins and bedrock knobs. In that case, their coincidence with the calculated subglacial water outlet locations would seem unlikely, and the initiation of ice-shelf channel morphology would not depend on subglacial water discharge.

### Evidence for actively evolving ramp shaped eskers

Our argument of reflectors A–C being eskers and not other (comparatively fixed) bedforms is supported by more evidence on the ice shelf: surface ridges dating from earlier times can be found there, implying past changes in the coupled systems.

The seeding of ice-shelf channels A–C at the grounding line means that their morphology can record grounding-line history as well as changes in sub-shelf melt plume dynamics as ice flow advects them towards the shelf front. At sites A and C, the surface ridges decay seawards to grade into the depressed surface of the ice-shelf channels downstream (Fig. 6) although we also find some isolated hills a few km downstream of the grounding line at both sites (e.g., Fig. 1b at location A2′). Ice-shelf channel A follows a streamline and extends to the ice-shelf front (Fig. 7a). About 45 km downstream of the grounding line, this channel is split by a surface ridge ∼10 km long and several hundred metres wide (Fig. 7b,c), which advects today and presumably has been advected to its present position from the grounding line. The advection time for its downstream end is 240 years and for its upstream end 175 years using present-day velocities^28^. We surveyed this ridge with ground-based radar. Cross-section R1–R1′ shows a basal channel directly under the ridge and a secondary basal channel laterally offset from it by ∼800 m. In contrast, cross-section R2–R2′ (farther upstream) shows a typical ice-shelf channel with a surface depression and a corresponding basal incision (Fig. 7d,e). We interpret these features as follows: More than 375 a ago, the ice-shelf channel was formed by a subglacial conduit exiting at the grounding line, which progressively developed a ramp-shaped esker and a local surface ridge there. About 240 ago, the surface ridge reached a critical height-to-width ratio, so that it was maintained in the ice shelf because bridging stresses prevent full relaxation to hydrostatic equilibrium^18^. Between 240 and 175 years ago, subglacial water had rerouted around the ramp-shaped esker and this is documented by the secondary basal channel in Fig. 7d. About 175 years ago, the surface ridge could no longer be supported mechanically on its landward end, perhaps due to a glacial outburst flood that eroded the esker. Other possibilities are that (1) subglacial drainage conditions changed in other ways to erode the esker more gradually or (2) changes in plume dynamics deepened and widened the ice-shelf channel sufficiently to eliminate the surface ridges. All scenarios considered here point to some past hydrological variability that is straightforwardly explained if the system involves an esker instead of other bedforms not closely related to subglacial conduit discharge.

In this interpretation, the surface ridges at sites A and C are currently below their critical threshold so they do not intrude far into the ice-shelf channels. But the isolated hills suggest that these ridges may soon be advected into the ice shelf. An end-member scenario is that the ramp-shaped esker at site A was completely removed by flooding 175 a ago and has since re-grown to its present size. This requires a sedimentation rate of ∼1.4 m a^−1^ as an upper limit, which is an order of magnitude higher than model estimates of sedimentation rates at melt-stream portals^36^. However, these estimates are geared towards ice-proximal fans and do not account for an upward-sloping bed interface, and critically depend on (unknown) subglacial sediment properties at the sites.

At site B, the basal disruption imaged by reflector B is offset from the surface ridge, and we lack ground-based radar data for examining its geometry along flow. In plan view, the corresponding ice-shelf channel is less developed compared to ice-shelf channels A and C and deviates from the ice-flow direction towards ice-shelf channel C. These observations suggest that the esker/conduit B began developing more recently and has been migrating eastward to reach its current position.

Numerous subglacial processes in scenario 3 require further investigation. Our mechanism for the formation of ramp-shaped eskers involves a reduction of water flow speed towards the grounding line, which has not been directly measured. Also, without access to the bed, we lack precise information about the current subglacial conduit arrangement and sediment transport regime on/near the eskers, which determine how their form continues to evolve. Since all three sites lack sediment sources on the surface, a negligible sediment delivery to the bed is expected from supraglacial melt-water streams^17^ even though melting is known to occur at the surface^43^.

It is noteworthy that our eskers are an order of magnitude larger than most eskers in deglaciated areas, which usually do not exceed 50 m in height^44^, although examples higher than 200 m exist^45^. Such difference may be explained by stability and preservation reasons, which consequently mean that the sizes of our eskers and deglaciated eskers are not directly comparable. As mentioned before, the ice flow and grounding line in the study area are thought to have been stable for millennia—such stability would promote the growth of large eskers. In contrast, eskers from the last-glacial record are often associated with retreating ice-sheet margins, which can limit their size. Moreover, our eskers are observed *in situ* in their formation environment, and confined by ice, which prevents sediment-flank slumping. If the grounding line retreats, rapid degradation by slumping and erosion would occur, especially as their sediments are probably weakly consolidated in the subaqueous environment; given enough time, a drastic height reduction is hence not inconceivable. These ideas seem to us consistent with the fragmented nature of esker networks from the Wisconsinan glaciation (e.g., Laurentide Ice Sheet), which typically consist of ridge segments with major gaps in between. Indeed, many of them may be the meagre remnant or core of originally much higher eskers.

Finally, the inferred ramp shape of our eskers has counterparts in the deglaciated landform record, notably eskers of type I–III described by Brennand^15^, which are thought to have formed by subaqueously terminating conduits. The Katahdin Esker in Maine, USA is a key example. It shows numerous ‘tadpole-shaped' segments, each several km long, that increase in size in the drainage direction. Hooke^17^ explained their origin by the same mechanism as proposed here and interpreted the corresponding ice margins had been stable for centuries. Our prediction (from Fig. 8) that each ramp decays rapidly over kilometres also suggests that their high topography should be rarely observed as a fraction of total esker length.

## Discussion

Our findings have several implications. First, the understanding of ice-shelf channel formation is now improved and more complicated than previously assumed. Three mechanisms have been suggested in other studies: first, ice-shelf channels can develop through meltwater plume flow combined with transverse variability in ice thickness^12^. Second, topographic highs in the bed upstream of the grounding line can locally thin the ice, and the incision is intensified seawards through oceanic-driven melting^11^,^12^,^46^. Third, ice-shelf channels can develop where subglacial melt-water channels exit from the Antarctic continent^6^,^9^,^10^. Here, we show that the last two mechanisms are probably linked. Subglacial conduits widen at the grounding line where the effective pressure becomes zero. This reduces water outflow speed and increases sedimentation, so that a ramp-shaped esker develops beneath the conduit if sufficient sediments are available. This means that large portions of the ice-shelf channel amplitude can already be determined landwards of the grounding line which has not been considered so far, and which is important because evidence of ice-shelf channels on ice-shelf stability is conflicting. Ocean melting beneath ice-shelf channels can protect the ice shelf from area-wide melting^11^,^47^, but channels may also weaken ice shelves through crevasse-formation^8^,^48^ or by opening-up entirely^8^,^49^. The ramp-shaped eskers may also locally pin the grounding line and thus stabilize the sheet-shelf system comparable to the self-stabilizing effect of sediment wedges^50^.

Second, evidence for channelized, subglacial water outlets supports the hypothesis that the meandering of ice-shelf channels seen in many ice shelves is an archive for the history of the subglacial hydrology in the respective drainage area^6^,^10^. In particular, conduit blockage by sediments is one candidate to explain why ice-shelf channels seem to converge in ice-shelf flow (i.e., channels tend to diverge at the grounding line over time Fig. 7a). Moreover, we discovered a surface ridge inside an ice-shelf channel archiving temporal variability in erosion/flushing and regrowth of the respective ramp-shaped esker farther upstream on the grounded ice.

Third, large ramp-shaped eskers upstream of the grounding line require stability of the subglacial hydrological system for their development. The Roi Baudouin Ice Shelf is a good candidate for this, because many ice-shelf channels extend from the grounding line to the ice-shelf front along flowlines, indicating temporal stability of the respective source at the grounding line over several hundreds of years. Analysis of an ice rise in that ice shelf suggests an even longer period of stability of the large-scale flow regime spanning several thousands of years^7^. Moreover, the build-up of sediments depends on the sediment supply and likely also on the basal conditions (hard versus soft bed) at the conduit's portal^33^. This may explain why such large bed disruptions have not yet been found elsewhere.

Fourth, the surface ridges and the relict ridge in the ice-shelf channel are key to our interpretation of the ramp-shaped esker formation. Therefore, our data provide field evidence for the evolution of a subglacial landform which is extensively investigated in other areas where ice-sheets have retreated^13^, but whose formation mechanisms are poorly understood^16^.

Fifth, the ramp-shaped esker/subglacial conduit system is clearly visible in our ground-based and airborne profiles about two ice thicknesses upstream of the grounding line. Their locations can be inferred from remote-sensing data, by aligning calculated water outlets with ice-shelf channels^10^, and possibly with characteristic surface ridges. Our observations, therefore, may provide comparatively easy access by surface drilling to a component of the Antarctic subglacial hydrological system which is so far essentially unknown.

In summary, we have discovered an actively evolving system of ramp-shaped eskers and subglacial conduits upstream of the grounding line of an Antarctic ice shelf. Seawards, ice-shelf channels are likely further incised by a buoyant melt-water plume forced by the meltwater exiting the conduit. The ice-shelf channel amplitudes, however, can already largely be determined by the height of the ramp-shaped esker. The latter develop through a generic process of conduit widening at the grounding line where creep closure is small. Diminished water outflow speed increases potential for sedimentation. Some ramp-shaped eskers are large enough to form corresponding ridges at the surface of the overriding ice. Depending on the height-to-width ratio, these ridges are preserved in the adjacent ice shelf and testify to an evolving subglacial hydrological system including rerouting of conduits and potentially flushing/eroding of the ramp-shaped eskers. Our findings improve the understanding of ice-shelf channel formation by exposing a novel link between subglacial hydrology and ramp-shaped esker formation impacting ice-shelf stability. Moreover, our data set is the first evidence for ramp-shaped esker formation beneath a contemporary ice sheet and sheds light on a subglacial landform that has been intensively studied in geomorphology for decades, but for which the formation mechanisms are only poorly constrained. Further research should be directed to more rigorously infer the existence and the type of sediments deposited by the subglacial conduit (e.g., with seismic surveys/hot-water drilling), which will facilitate quantitative modelling of sediment transport at the basal boundary of ice sheets.

## Methods

### Airborne and ground-penetrating radar

Ground-based and airborne radars image the thickness and internal stratigraphy of ice by measuring the traveltimes of reflections from actively emitted electromagnetic waves in the MHz range. Reflections originate from changes in ice density, ice acidity or a preferred orientation of the ice crystals^51^. The data presented in Fig. 1c and Supplementary Fig. 2 have been collected with a pulsed, airborne radar at a centre frequency of 150 MHz and were previously discussed^29^. We refer to this publication for details of the data collection and processing. The ground-based radar has resistively loaded dipole antennas with a nominal centre frequency of 10 MHz^52^. Geolocation of the radar traces was done using a geodetic GNSS receiver attached to the radar's receiver and collecting at 1 s intervals (Surface topography from GNSS and TanDEM-X). The radar was towed at approximately 3.6 m s^−1^ and traces were horizontally stacked to common postings (∼12 m) during the post-processing. After bandpass filtering (frequency range between 3 and 9 MHz), the data were migrated to account for off-angle reflections from sloped interfaces (e.g., the lateral walls of the subglacial conduit/ice-shelf channels) using Kirchoff-Depth migration implemented in the open-source software Seismic Unix. The required radio-wave velocity model varies with depth using a depth–density parameterization^53^ and a density–velocity relation^54^. The grounding-zone of Roi Baudouin Ice Shelf is characterized by an extensive blue-ice belt so that the radio-wave velocity is close to the pure-ice velocity (1.68·10^8^ m s^−1^) everywhere. We, therefore, chose the parameters (surface density and densification length) of the depth-density function so that the equivalent firn-air content is below 1 m. Picking of internal reflections and 3D-visualization is done using seismic interpretation software (OpenDtect).

Bandpass filtering/migration causes ringing near the strong reflections originating from the bed and the upper surface of the subglacial conduit. These artefacts obstruct analysis of the wavelet's phase structure, which is therefore done using the raw data only. We find no phase reversal for both reflections types, indicating a transition from an optically less dense (with faster radio-wave velocity) to an optically denser (with a slower radio-wave velocity) background medium. This is consistent with a water and or sediment-filled conduit which is overlain by (less-dense) ice.

### Surface topography from GNSS and TanDEM-X

The kinematic GNSS data were collected with a dual-frequency GNSS receiver attached to the radar's receiver. We estimated the position of the moving station at 1 Hz sampling rate using the GAMIT/GLOBK v10.5 software^55^. The positions are calculated relative to a base station situated on the ice shelf considered as static. The coordinates of the base station are determined daily, using the Precise Point Positioning Atomium software^56^. We neglected the daily horizontal movement of the base station which is less than 1 m. The vertical displacement of the ice-shelf surface by tides is less than 1 m in this area and does not impact the interpretation of the radar data done here.

The satellite-derived surface elevations stem from TanDEM-X, a radar satellite pair imaging the ice-sheet surface using the X-band with small signal penetration into the surface. The elevation model is mosaicked out of 40 TanDEM-X single look complex scenes acquired in austral winter 2013. Scenes were processed individually using the SARscape software. The processing includes coregistration with the CryoSat-2 surface elevation^57^, filtering of the differential interferogram, unwrapping, phase re-flattening and a final geo-referencing. The final digital elevation model is gridded to 10 m cells with an estimated relative vertical accuracy of better than 1 m based on the standard deviation of the overlapping scenes.

### Data availability

All data are available from the authors on request.

## Additional information

**How to cite this article:** Drews, R. *et al*. Actively evolving subglacial conduits and eskers initiate ice shelf channels at an Antarctic grounding line. *Nat. Commun.* **8,** 15228 doi: 10.1038/ncomms15228 (2017).

**Publisher's note**: Springer Nature remains neutral with regard to jurisdictional claims in published maps and institutional affiliations.

## Supplementary Material

Supplementary Information Supplementary Notes, Supplementary Table, Supplementary Figures and Supplementary References
