# Supplementary material for: Actively evolving subglacial conduits and eskers initiate ice shelf channels at an Antarctic grounding line
Source: Nat Commun. 2017 May 9;8:15228. doi: 10.1038/ncomms15228 (PMC5482720; doi:10.1038/ncomms15228)
Supplement: Supplementary Information — Supplementary Notes, Supplementary Table, Supplementary Figures and Supplementary References [file ncomms15228-s1.pdf]

Supplementary Note 1: Locating the grounding line

The grounding line is the point where ice loses contact with the bed and becomes afloat. This point can be mapped with different methods such as satellite altimetry [1], seismics [2], break-in-slope [3], repeat kinematic GPS profiling [4], ground-based radars [5], and satellite interferometry [6]. Here, we use the last two techniques and show that the radar-cross sections A1-A1', EuA-EuA' and C1-C1' (and the reflectors A-C seen therein) are upstream of the tidal flexure zone as seen from satellites. We find some evidence that ocean water may intrude upstream of that boundary forming an estaurine setting observed elsewhere [2,4].

We map the landward limit of the tidal flexure zone with satellite-based interferometric synthetic aperture radar (InSAR) using data from Sentinel 1A collected in 2016, ALOS-PALSAR collected in 2007, and from the European-Remote-Sensing satellites (ERS) 1/2 with acquisitions during their tandem phase in 1996. Coregistration and differencing of two scenes yields an interferogram which is color-coded by lines of constant phase differences (a.k.a. fringes). Fringes are a function of satellite geometries, surface velocities [7,8,9] and surface topography [10]. A flattened interferogram, contains mixed contributions of topography and velocity. Using the TanDEM-X elevation model, we correct for the topography.

For the ERS acquisitions, no second interferogram is available so that we cannot strictly separate between vertical and horizontal displacement in the topographically corrected interferogram. However, due to the steep look angle of the ERS-satellites, the interferogram is most sensitive to the vertical displacement of the ice shelf by tides which causes a dense fringe pattern signifying the grounding-zone [6]. We pick the landward limit of this pattern as a satellite-based estimate for the grounding-line location in 1996. We use a similar processing scheme for the Sentinel 1A and the ALOS- PALSAR images. In those cases, three coherent overflights are available and we calculate differential interferograms. Because the steady component of the horizontal ice flow is cancelled, this makes the grounding zone more prominent. Supplementary Fig. 1 shows the differential interferogram from 2016 alongside the picked grounding lines from 2007 and 1996. The three estimates agree in many areas and differences are often within a few hundred meters.

Next, we consider two ground-penetrating radar profiles across the grounding zone collected in 2016 (GL-GL', A2-A2' located in Fig. 1). The grounding line can be determined by looking for abrupt changes in the amplitude of the basal reflection indicating the transition from a rough/diffuse ice--bed reflector to the bright/specular ice-ocean interface. Such abrupt changes are often found [5], although a more gradual transition in basal reflectivity can also occur (e.g. when basal ice is enriched with debris [4]). For the profile GL-GL', basal reflectivity abruptly changes near kilometer 2 which is about 1 km upstream of the landward limit of tidal flexure (Fig. 3). This abrupt change hints to a water layer at the ice-bed interface which may be of continental and/or oceanic origin. For the latter, observations are similar to findings near an embayment of the Whillans Ice Stream, West Antarctica, where ocean water can penetrate upstream of the tidal flexure zone through tidal pumping forming an estuary with two-way water exchange [11,2,4]. Alternatively, we could be seeing subglacial water which is routed towards the ocean.

The profile A2-A2' (Figs. 2 and 5) is collected in an area with extensive surface (and potentially basal) crevassing making the analysis more complicated due to out of plane reflections [12]. The profile is dominated by the strong reflector A. At larger depths, we can trace another reflector along-flow which we identify as the ice--bed interface because it links with the ice-bed interface of across-flow profiles farther upstream. However, because the radar wave does likely not penetrate through reflector A, the origin of the radar reflector remains unclear and we tentatively attribute it to an off-angle reflection from a rough bed. This is supported by the fact that a similar reflector is not evident in the neighboring radar section A3-A3' which is located more centrally on top of reflector A. The disappearance of the off-angle reflector in A2-A2' after 1.8 kilometers marks in this context the grounding line. This location is only about 300 m downstream from the landward limit of tidal flexure. We receiver no reflections from the floating ice shelf in this area farther downstream.

Because the tidal flexure zones from 1996, 2007 and 2016 are very similar, we can exclude a systematic grounding-line migration in that time interval. This accords with modelling results showing that the grounding line of the Roi Baudouin Ice Shelf is topographically controlled and is unlikely to retreat even for scenarios with high basal melt rate [13]. Therefore, it is most likely that the radar-cross sections A1-A1', EuA-EuA' and C1-C1' (and the reflectors A-C seen therein) are completely on grounded ice. Ocean water may, perhaps episodically, infiltrate upstream of the tidal flexure zone, which we discuss in more detail in Section 3.2.

Supplementary Note 2: Evolution of subglacial conduits at the grounding line

We base our model of the subglacial conduits on the basic physics developed by [14] and [15] where the conduit size is determined by a balance between ice melting at the conduit walls (the heat being provided from the turbulent water flow) and creep closure (forced by the overburden ice pressure). Close to the grounding line, the magnitude of the water flux through the conduit Q is largely determined by the catchment basin farther upstream and, neglecting additional water input from melting at the channel walls near the grounding line, we consider the flux as constant. The model only considers the evolution of the conduit's cross-sectional area (S) and the hydraulic potential gradient (Φ). In a coordinate system where x is along-flow and z in the vertical, a relationship between these two quantities follows from parametrizing the turbulent drag on the walls of the conduit,

$$\phi = \phi_0 + \frac{\partial N}{\partial x} = K \frac{Q^2}{S^{5/2}}. \qquad (1)$$

Here,

$$K = \rho_w f(\pi + 2)/(2^{5/2}\sqrt{\pi})$$

is a constant (using the Darcy-Weisbach parameterization with water density ρ_w, friction parameter f, and a semi-circular cross section). The potential gradient Φ consists of two component: (i) the background potential which is given by the ice geometry

$$\phi_0 = -\rho_i g \frac{\partial s}{\partial x} - (\rho_w\text{-}\rho_i) g \frac{\partial b}{\partial x}$$

(where $z = s$ and $z = b$ are the ice surface and bed elevations), and (ii) the effective pressure gradient, $\frac{\partial N}{\partial x}$, where $N = \rho_i - \rho_w$ is the difference between water (ρ_w) and ice-overburden pressure (ρ_i) inside the conduit. The effective pressure gradient becomes important near to the grounding line where the effective pressure itself must be zero if the ice floats.

A steady-state balance of wall melting and creep closure at the conduit walls requires

$$u_b \frac{\partial S}{\partial x} = \frac{Q}{\rho_i L}\left(\phi_0 + \frac{\partial N}{\partial x}\right) - \hat{A} S N^n \qquad (2)$$

where L in the first term of the right-hand side is the latent heat describing the melting, and $\hat{A} = \frac{2A}{n^n}$ depicts the creep closure using Glen's flow law coefficient for exponent $n = 3$. The advection term on the left-hand side (with ice sliding speed u_b), like the effective pressure gradient, becomes important near the grounding line. We apply a simplified, fixed geometry (a flat bed at a depth of b = -700 m, and a constant surface slope $\frac{\partial s}{\partial x}$ = 0.016, Fig. 8A) approximating the tributary Ragnhild glaciers of the Roi Baudouin Ice Shelf [16,17]. This yields a constant potential gradient $\phi_0 \approx$ 180 Pa m^{-1}.

Using the parameters listed in Supplementary Table 1 for solving eqs. (1) and (2) subject to the boundary condition N = 0 at the grounding line results in the steady-state conduit area in Fig. 8B. Neglecting the advection term (left-hand term in eq. (2), we find that the conduit's cross-section grows substantially in a small boundary region near the grounding line which can be conceptually understood as follows: If ice floats, the effective pressure is zero and no creep closure occurs. Melting at the channel walls, on the other hand, persists and causes the cross-sections to grow to infinity in the absence of ice advection (Fig. 8B). A similar divergence also occurs at water outlets of alpine glaciers [18]. However, including the advection of a smaller conduit cross-sections from upstream limits their growth at the grounding line.

A more detailed analysis of eqs. (1) and (2) shows that the conduit cross-section at the grounding line is given by

$$S_0 \approx C {u_b}^{-\frac{3}{14}} {\phi_0}^{-\frac{3}{14}} Q^{\frac{6}{7}} \qquad (3)$$

where $C = 1.4 K^{\frac{2}{7}} {\rho_i}^{-\frac{2}{7}} L^{-\frac{2}{7}} \hat{A}^{-\frac{1}{14}}$ is a constant. The cross-section is larger for a larger discharge (which provides more dissipative power), smaller ice speeds (which allow more time for the conduit to grow), and smaller surface slopes (which allow the region of low effective pressure to extend farther upstream). The results shown in Fig. 8C-D consider an upper estimate (with a large subglacial discharge, Q = 100 m^3 s^{-1}, and low basal sliding, u_b = 1 m a^{-1}) and a lower estimate (Q = 10 m^3 s^{-1} and u_b = 300 m a^{-1}). These combinations bracket the observed surface velocities [9] and the estimated total subglacial melt water flux across the grounding line ($\sim$ 60 m^3 s^{-1}) using a hybrid ice-stream/ice-shelf model [19].

Although the conduit predicted by this model grows substantially near the grounding line, the absolute magnitude (for the parameter-set considered here) is smaller than our observations. This is mainly because the flattening of the hydraulic potential also reduces the outflow water speed together with the melting at conduit walls (Fig. 8C, D). Additionally, the assumption of isothermal ice close to the melting point becomes increasingly violated closer to the ice-sheet surface where the ice is colder. The conduit-widening described here is, therefore, not the only mechanism which needs to be considered.

Intrusion of ocean water into the mouth of the conduit and subsequent mixing with the subglacial discharge, may amplify the melting [20] and lead to a larger cross-section than predicted by eq. (3). Such intrusion is possible because outflow speed of the subglacial meltwater decrease near the grounding line. With a depth-averaged ice velocity of 300 m a^{-1} an additional melt rate stemming from the ocean of $\sim$10 m a^{-1} is needed to grow a conduit

with a 100 m radius over a horizontal distance of 3 km. However, even these increased melt rates do not accord with our observations of surface ridges above the conduits because wall melting principally lowers the ice surface. We, therefore, discuss in the main text the impact of sedimentation at the conduit's portal. For the latter, the reduction of water outflow speed due to the conduit widening predicted by our simplified model (Fig. 8D) is critical.

ρ_w = 1000 kg m^{-3}	ρ_s = 1028 kg m^{-3}
ρ_i = 910 kg m^{-3}	A = 2.4 · 10^{-24} s^{-1} Pa^{-3}
L = 3.3 · 10^{5} J kg^{-1}	n =3
g = 9.81 m s^{-2}	f = 0.2

Supplementary Table 1: Summary of model parameters ρ_w (density of fresh water), ρ_s (density of ocean water), ρ_i (density of ice), A (flow parameter), L (latent heat of fusion), n Glen's index, and g (gravitational acceleration).

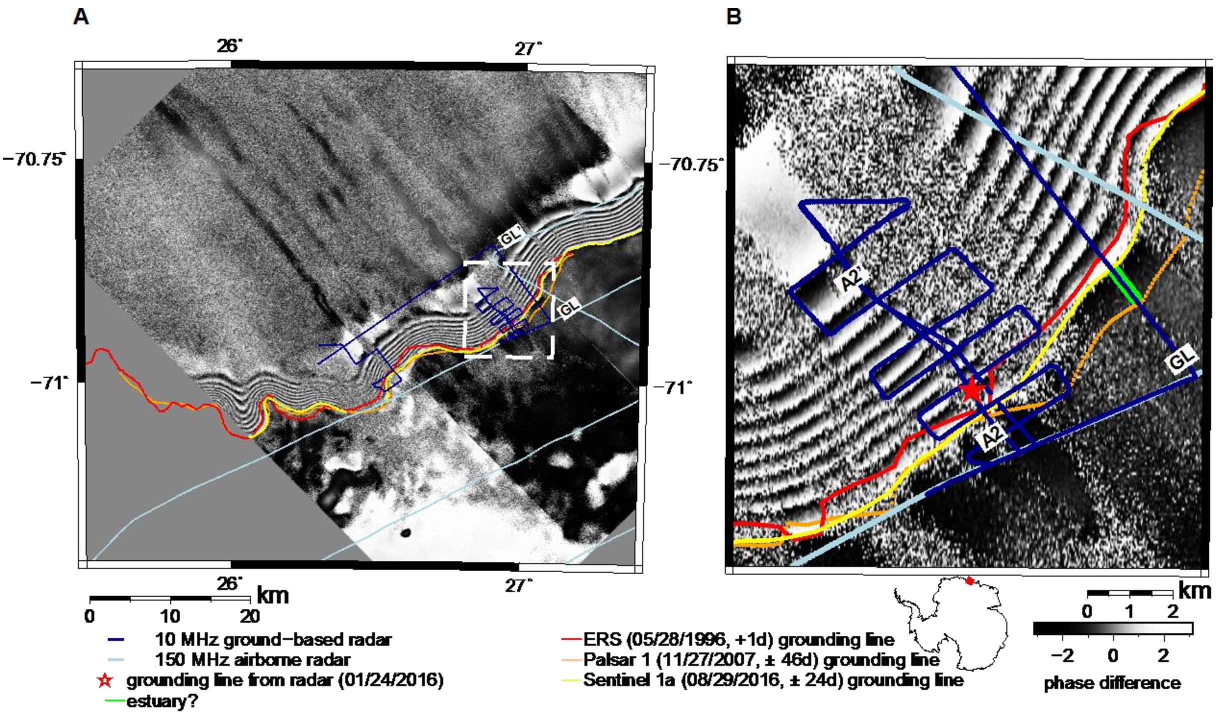

Supplementary Figure 1: A: Landward limit of the tidal flexure zone from radar interferometry in 1996, 2007 and 2016 with double-differenced Sentinel 1A interferogram (2016) shown in the background. The white box delineates the area zoomed in in B. The The vertical displacement by tides is reflected by the dense fringe pattern, separating the grounded ice from the floating ice shelf and we find no significant temporal variations between 1996 and 2016. The ground-based radar profiles A2-A2' (Fig. 2) and GL-GL' (Fig. 4) are used to infer the grounding line with radar (red star). In GL-Gl' some water, which maybe of oceanic or continental origin, is found upstream of the tidal flexure zone indicating potential for an estaurine grounding-zone (green line).

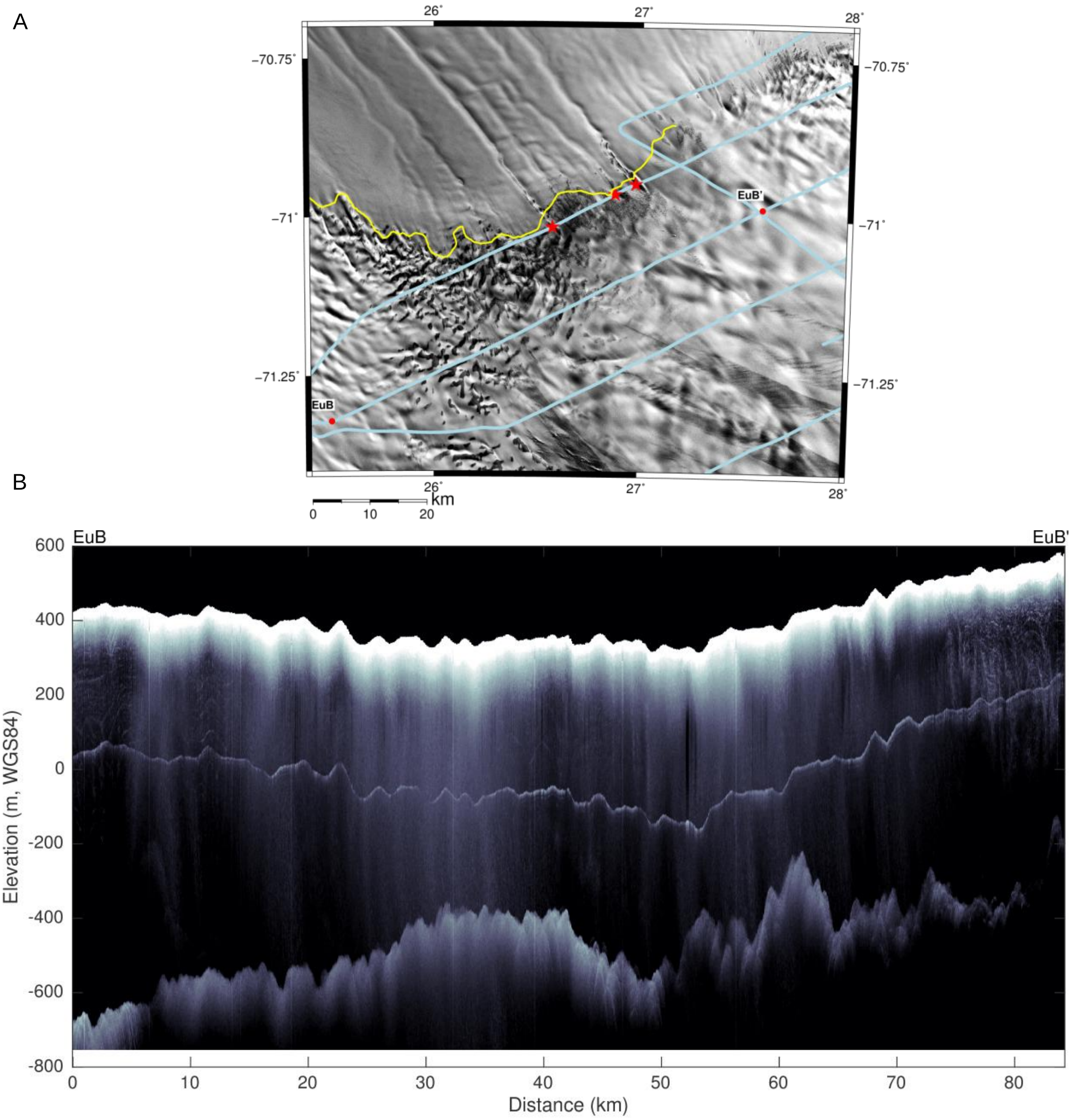

Supplementary Figure 2: The airborne radar profile EuB-EuB' is located about 15 km upstream of the reflectors A-C (Fig. 1) (A) and shows no comparable radar returns (B).

Supplementary References

[1] H. A. Fricker et al. “Mapping the grounding zone of the Amery Ice Shelf, East Antarctica using InSAR, MODIS and ICESat”. Antarctic Science 21.05 (2009), p. 515.

[2] H. J. Horgan et al. “Sediment deposition at the modern grounding zone of Whillans Ice Stream,West Antarctica”. Geophysical Research Letters 40.15 (2013), pp. 3934–3939.

[3] R. Bindschadler et al. “Getting around Antarctica: new high-resolution mappings of the grounded and freely-floating boundaries of the Antarctic ice sheet created for the International Polar Year.” The Cryosphere 5.3 (2011), pp. 569–588.

[4] K. Christianson et al. “Basal conditions at the grounding zone of Whillans Ice Stream, West Antarctica, from ice-penetrating radar”. Journal of Geophysical Research: Earth Surface 121.11 (2016), pp. 1954–1983.

[5] K. Matsuoka et al. “Radar characterization of the basal interface across the grounding zone of an ice-rise promontory in East Antarctica”. Annals of Glaciology 53.60 (2012), pp. 29–34.

[6] E. Rignot, J. Mouginot, and B. Scheuchl. “Antarctic grounding line mapping from differential satellite radar interferometry”. Geophysical Research Letters 38.10 (2011), p. L10504.

[7] E. Rignot, J. Mouginot, and B. Scheuchl. “Ice flow of the Antarctic ice sheet.” Science 333.6048 (2011), pp. 1427–1430.

[8] N. Neckel et al. “Basal melting at the Ekström ice shelf, Antarctica, estimated from mass flux divergence”. Annals of Glaciology 53.60 (2012), pp. 294–302.

[9] S. Berger et al. “The control of an uncharted pinning point on the flow of an Antarctic ice shelf”. Journal of Glaciology 62.231 (2016), pp. 37–45.

[10] R. Drews et al. “A spatially adjusted elevation model in Dronning Maud Land, Antarctica, based on differential SAR interferometry”. IEEE Transactions on Geoscience and Remote Sensing 47.8 (2009), pp. 2501–2509.

[11] R. T. Walker et al. “Ice-shelf tidal flexure and subglacial pressure variations”. Earth and Planetary Science Letters 361 (2013), pp. 422–428.9

[12] R. W. Jacobel et al. “Morphology of basal crevasses at the grounding zone of Whillans Ice Stream, West Antarctica”. In: Annals of Glaciology 55.67 (2014), pp. 57–63.

[13] L. Favier et al. “Dynamic influence of pinning points on marine ice-sheet stability: a numerical study in Dronning Maud Land, East Antarctica”. The Cryosphere 10.6 (2016), pp. 2623-2635.

[14] H. Röthlisberger. “Water pressure in intra-and subglacial channels”. J. Glaciol. 11.62 (1972), pp. 177–203.

[15] J. F. Nye. “Water flow in glaciers: Jökulhaups, tunnels, and veins”. Journal of Glaciology 76.17 (1976), pp. 181–207.

[16] D. Callens et al. “Transition of flow regime along a marine-terminating outlet glacier in East Antarctica”. The Cryosphere 8.3 (2014), pp. 867–875.

[17] D. Callens et al. “Mass balance of the Sør Rondane glacial system, East Antarctica”. Annals of Glaciology 56.70 (2015), pp. 63–69.

[18] G. W. Evatt. “Röthlisberger channels with finite ice depth and open channel flow”. Annals of Glaciology 56.70 (2015), pp. 45–50.

[19] F. Pattyn. “Antarctic subglacial conditions inferred from a hybrid ice sheet/ice stream model”. Earth and Planetary Science Letters 295.3-4 (2010), pp. 451–461.

[20] A. Jenkins. “Convection-Driven Melting near the Grounding Lines of Ice Shelves and Tidewater Glaciers”. Journal of Physical Oceanography 41.12 (2011), pp. 2279–2294.
